# Association Between Problematic Online Gaming and Subsequent Psychotic Experiences in Adolescents: A Birth Cohort Study

**DOI:** 10.1192/bjo.2024.216

**Published:** 2024-08-01

**Authors:** Zui Narita, Syudo Yamasaki, Satoshi Yamaguchi, Shuntaro Ando, Atsushi Nishida

**Affiliations:** 1 National Center of Neurology and Psychiatry, Kodaira, Japan; 2 Tokyo Metropolitan Institute of Medical Science, Setagaya, Japan; 3 University of Tokyo, Bunkyo, Japan

## Abstract

**Aims:**

There is still little information available on the negative impact of online activities on psychotic experiences. This limitation is further compounded for online gaming, where even a beneficial impact has been suggested via the evocation of positive emotions. We aimed to examine how problematic online gaming (POG) is associated with subsequent psychotic experiences in adolescents.

**Methods:**

This birth cohort study employed randomly sampled adolescents born between September 2002 and August 2004. The eligibility criterion was those who did not have psychotic experiences at age 14. We analyzed the association between POG at age 14 and subsequent psychotic experiences at age 16. Adolescents were categorized into the no, low, and high POG groups based on the behaviors and emotions related to online gaming at age 14. Missing data were handled using random forest imputations.

**Results:**

A total of 1722 adolescents without psychotic experiences at age 14 were analyzed. At age 16, 55 adolescents exhibited psychotic experiences, while 225 showed potential psychotic experiences. Compared with the no POG group, a higher risk of psychotic experiences was shown in both the low (RR 1.93, 95% CI 1.74–2.15) and high (RR 2.81, 95% CI 2.50–3.15) POG groups. Findings were consistent when analyzing potential psychotic experiences.

**Conclusion:**

POG appears detrimental to the development of psychotic experiences in adolescents. Our findings provide public health implications in the context of policymaking.

